# Control of Stimuli Sensitivity in pH-Switchable LCST/UCST-Type Thermosensitive Dendrimers by Changing the Dendrimer Structure

**DOI:** 10.3390/polym14122426

**Published:** 2022-06-15

**Authors:** Chie Kojima, Yunshen Fu, Mamiko Tamaki

**Affiliations:** Department of Applied Chemistry, Graduate School of Engineering, Osaka Prefecture University, 1-1, Gakuen-cho, Naka-ku, Sakai 599-8531, Osaka, Japan; mf105117@st.osakafu-u.ac.jp (Y.F.); tamakimamiko@gmail.com (M.T.)

**Keywords:** dendrimer, phenylalanine, zwitterionic, pH-sensitive, thermosensitive

## Abstract

Stimuli-sensitive materials, such as pH- and temperature-responsive polymers, are useful as smart materials. Phenylalanine (Phe)-modified polyamidoamine (PAMAM) dendrimers with succinic acid termini, PAMAM-Phe-Suc, have been reported as unique pH-switchable lower critical solution temperature (LCST)-/upper critical solution temperature (UCST)-type thermosensitive polymers. Regulating the phase transition behavior of dendrimers is important for their applications. This study investigated the relationship between the dendrimer structure and stimuli sensitivity. Phe-modified PAMAM dendrimers with cyclohexanedicarboxylate termini (PAMAM-Phe-CHex) and sulfonate termini (PAMAM-Phe-SO_3_Na) were synthesized. The temperature-dependent transmittance of these aqueous dendrimer solutions was examined at various pH values. PAMAM-Phe-CHex with Phe at all termini (PAMAM-Phe64-CHex) demonstrated a broad UCST-like phase transition at pH 7.0 but lacked an LCST-type phase transition. PAMAM-Phe-CHex with ≤ 27 Phe residues showed both LCST- and UCST-like phase transitions at different pH values, but the phase transition was broad. PAMAM-Phe-SO_3_Na showed both LCST- and UCST-type phase transitions at different pH values, and the transition temperature increased as the bound Phe number decreased. Thus, the phase transition behavior of PAMAM-Phe-SO_3_Na dendrimers can be regulated by varying the Phe/PAMAM ratios.

## 1. Introduction

Smart materials, such as temperature-responsive and pH-sensitive polymers, have attracted significant interest in various fields [1,2]. Poly(*N*-isopropylacrylamide) (PNIPAM) is a typical example of a lower critical solution temperature (LCST)-type thermosensitive polymer [3,4]. Poly(*N*-acryloyl glycinamide) with amide bonds and poly(sulfobetaine methacrylate) with zwitterionic groups are upper critical solution temperature (UCST)-type thermosensitive polymers [5,6,7]. LCST-type thermosensitive polymers are soluble at low temperatures but are insoluble above the LCST. In contrast, UCST-type thermosensitive polymers are insoluble at low temperatures but are soluble above the UCST. Block copolymers bearing both LCST and UCST thermosensitive segments were designed as unique polymers exhibiting both LCST- and UCST-type thermosensitive behaviors [8]. It was reported that the LCST-type thermosensitive behaviors of PNIPAM in water were changed into the UCST-type in a binary aqueous solution containing alcohol and DMSO [9]. Some polymers with the LCST/UCST switching behaviors by changing solvents, like PNIPAM, were also reported [10,11,12]. Besides, the addition of salt induced the switching of the LCST/UCST behaviors of these LCST/UCST diblock polymers [5,6,13,14,15]. Due to the fact that the control of these phase transition temperatures is important for the application of thermosensitive polymers, various kinds of polymers have been studied [5,6,7].

**Figure 1:**
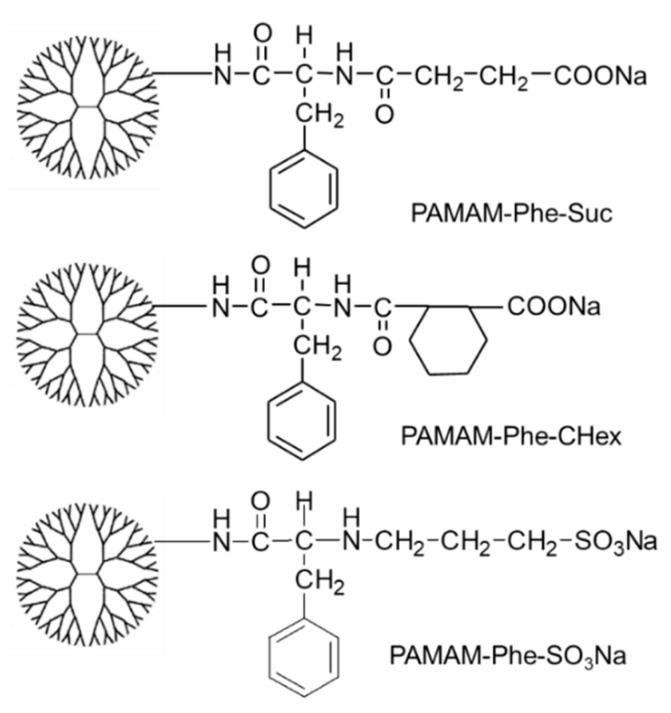
Dendrimer structures of PAMAM-Phe-Suc, PAMAM-Phe-CHex and PAMAM-Phe-SO_3_Na.

Dendrimers are highly branched polymers synthesized via a stepwise reaction. The molecular weight, shape, and size of dendrimers are highly controllable. Since dendrimers can load several types of molecules via attachment to their terminal groups or encapsulation into their interiors, they are useful as multifunctional nanocarriers and smart materials [16,17,18,19]. Stimuli-responsive dendrimers have previously been reported [20]. For example, LCST-type thermosensitive dendrimers have been produced via modification of dendrimers with various compounds, including oligo(ethylene glycol), *N*-isopropyl groups, and elastin-like peptides, which are useful for drug delivery and substance separation [20,21,22,23,24]. However, there are limited reports of UCST-type thermosensitive dendrimers. The carboxylate-terminal phenylalanine (Phe)-modified polyamidoamine (PAMAM) dendrimers showed UCST-type thermosensitivity at pH 5 [25], whereas the amino-terminal Phe-modified PAMAM dendrimers showed LCST-type thermosensitivity at pH 7.4 [26]. The zwitterionic structure of the carboxylate-terminal Phe-modified dendrimers may induce UCST-type thermosensitive behavior [25]. Two carboxylate-terminal Phe-modified PAMAM dendrimers (PAMAM-Phe-Suc and PAMAM-Suc-Phe), synthesized by changing the reaction order of succinamic anhydride and Phe, demonstrated different pH and temperature sensitivities. Interestingly, PAMAM-Phe-Suc exhibited both UCST- and LCST-type thermosensitivity at pH 5.5 and 4, respectively [27]. A sulfonate-terminal dendrimer (PAMAM-Phe-SO_3_Na) also exhibited UCST- and LCST-type thermosensitivity modulated by pH [27]. The control of stimuli sensitivity in these pH-switchable LCST/UCST-type thermosensitive dendrimers is important for their application. Hydrophobicity is known to significantly affect thermosensitivity [5,6,7]. This study investigated the relationship between the dendrimer structure and stimuli sensitivity in unique pH-switchable LCST/UCST-type thermosensitive dendrimers. Phe-modified PAMAM dendrimers with cyclohexanedicarboxylate termini (PAMAM-Phe-CHex) were synthesized. Moreover, we varied the Phe/PAMAM ratio in PAMAM-Phe-CHex and PAMAM-Phe-SO_3_Na to regulate their stimuli-responsive properties (Figure 1). Their stimuli-responsive properties were investigated and compared to PAMAM-Phe-Suc. Our results provide a basis for the molecular design of pH-switchable LCST/UCST-type thermosensitive dendrimers.

## 2. Materials and Methods

### 2.1. Materials

PAMAM-Phe-Chex and PAMAM-Phe-SO_3_Na, bearing Phe at all dendrimer termini, were synthesized as previously reported [27,28]. Briefly, *N*-(*tert*-butoxycarbonyl)-l-phenylalanine (Boc-Phe) was reacted with 1.3 equivalents of *N*,*N′*-dicyclohexylcarbodiimide (DCC), 1.2 equivalents of *N*-hydroxysuccinimide (NHS), and 1.5 equivalents of triethylamine (TEA) in *N*,*N*-dimethyl formamide (DMF, anhydrous) for 4 h on ice. Then, PAMAM dendrimer with an ethylenediamine core of a generation 4 (G4) dissolved in dimethyl sulfoxide (DMSO, anhydrous) was added at a molar ratio of 96/1 to Boc-Phe/PAMAM. The reaction mixture was stirred for 2 days at room temperature. These compounds were purified by dialysis (MWCO 2k) using methanol. After drying under vacuum, PAMAM–(Boc-Phe) was obtained. Then, the dendrimer was dissolved in trifluoroacetic acid (TFA) and reacted for 4 h on ice to remove the Boc group. Finally, excess 1,3-propane sultone dissolved in acetonitrile or cyclohexanedicarboxylic anhydride powder was reacted with all dendrimer termini in 125 mM NaHCO_3_ buffer. Similarly, PAMAM-Phe-Chex and PAMAM-Phe-SO_3_Na, with different Phe residues, were synthesized by varying the Boc-Phe/PAMAM ratio (Table 1).

### 2.2. Characterization

Proton nuclear magnetic resonance (^1^H-NMR) spectra were recorded using a 400 MHz instrument (JEOL Ltd., Tokyo, Japan). Fourier transform-infrared (FT-IR) spectra were recorded by using JASCO FTIR 4600 spectrometer (Jasco Inc., Tokyo, Japan). A powder dendrimer sample was sandwiched between KBr plates for the FT-IR measurement.

Dendrimer-containing solutions were prepared using 100 mM buffers (dendrimer 1 mg/mL, buffer 20 mM) at different pH values. Phosphate (pH 6 and higher) and acetate (pH 3.5–6) buffer solutions were prepared. Phosphoric acid was used to adjust the pH to <3.5. The temperature-dependent transmittance at 500 nm was measured with a 1.0 °C/min heating speed using a Jasco Model V-630 UV/Vis spectrophotometer equipped with an ETC-717 (Jasco Inc.). The dendrimer solutions were prepared using heavy water and the temperature-dependent transmittance curves were also measured.

## 3. Results and Discussion

### 3.1. Synthesis of Various Anionic Terminal Dendrimers Bearing Phe

**Figure 2:**
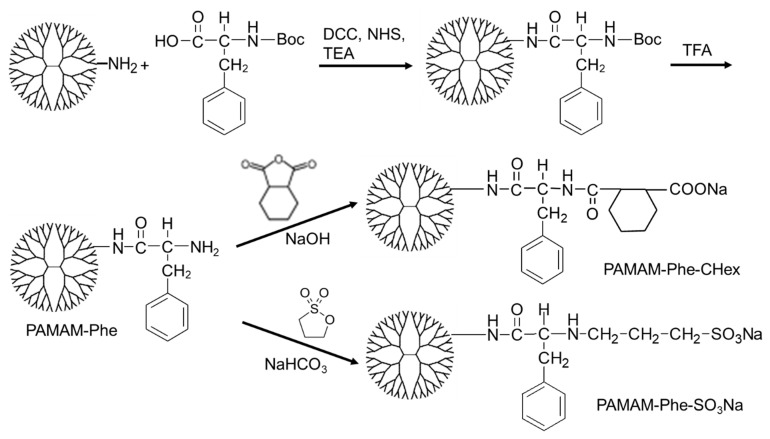
Synthetic scheme of PAMAM-Phe-CHex and PAMAM-Phe-SO_3_Na.

PAMAM-Phe-Chex and PAMAM-Phe-SO_3_Na were synthesized according to our previous reports [27,28] (Figure 2). Briefly, the PAMAM G4 dendrimer was reacted with excess Boc-Phe using condensation agents. The bound number of Boc-Phe was changed by varying the Phe/PAMAM ratio (Table 1). After deprotection with TFA, excess cyclohexanedicarboxylic anhydride or 1.3-propane sultone was reacted with all termini of the dendrimer in an alkaline solution. The synthesized dendrimers are presented in Table 1. The average bound number of Phe was evaluated from the average integral ratios of the signals at 7.0–7.4 ppm, 4.2 ppm, and 1.2 ppm (Boc-Phe) to 2.2 ppm (dendrimer) in the ^1^H-NMR spectra of PAMAM-(Boc-Phe) (see Supplementary Figures). The removal of Boc from the dendrimer was confirmed by the disappearance of the Boc signal at 1.2 ppm (see Supplementary Figures). The bound number of CHex was evaluated from the integral ratio of the signals at 1.1–1.9 ppm (CHex) to 7.0–7.4 ppm (Phe) in the ^1^H NMR spectra (see Supplementary Figures). The bound number of propane sultone (SO_3_Na) was evaluated from the integral ratio of the signals at 1.8 ppm (center methylene in propane sultone) to 7.0–7.4 ppm (Phe) in the ^1^H NMR spectra (see Supplementary Figures). Since 1,3-propane sultone could react with both primary and secondary amines, the bound number of SO_3_Na possibly exceeded 64 dendrimer termini. The FT-IR spectroscopy of these dendrimers was carried out. The FT-IR spectra of PAMAM-Phe-CHex and PAMAM-Phe-SO_3_Na contained amide signals (1550 cm^−1^, 1650 cm^−1^, around 3300 cm^−1^, and around 3500 cm^−1^) derived from the PAMAM dendrimer and mono-substituted benzene signals (700 cm^−1^ and 750 cm^−1^) derived from Phe [29]. Sulfonate signals (1040 cm^−1^ and 1180 cm^-1^) were observed in the FT-IR spectra of PAMAM-Phe-SO_3_Na [29]. A signal at 2940 cm^−1^, derived from cyclohexane of CHex, was enlarged in the FT-IR spectra of PAMAM-Phe-CHex [29]. Thus, these results indicated that different terminal Phe-modified PAMAM dendrimers were synthesized.

### 3.2. Thermosensitivity at Different pH Values

**Figure 3:**
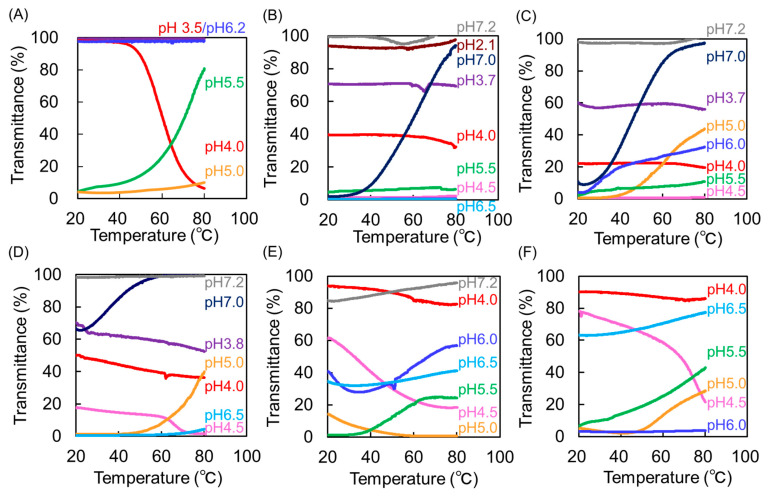
Temperature-dependent transmittance curves of aqueous solutions containing carboxylate-terminal Phe-modified dendrimer with different Phe/PAMAM ratios and different terminal groups at different pH values (1 mg/mL). (**A**) PAMAM-Phe56-Suc was referred to our previous paper [27]. (**B**) PAMAM-Phe-64-CHex, (**C**) PAMAM-Phe46-CHex, (**D**) PAMAM-Phe35-CHex, (**E**) PAMAM-Phe27-CHex, and (**F**) PAMAM-Phe16-CHex.

We examined the temperature-dependent transmittance of the synthesized Phe-modified dendrimers at different pH values. Stimuli-responsive behaviors of these dendrimers were compared to those of PAMAM-Phe56-Suc and PAMAM-Phe57-SO_3_Na which were previously reported [27]. First, we attempted to regulate the stimuli sensitivity of the carboxylate-terminal Phe-modified dendrimers. Figure 3A demonstrates the thermosensitivity of PAMAM-Phe56-Suc at different pH values. The UCST and LCST were defined as the temperature at which the transmittance was 50%. The dendrimer solutions at pH 6.0 or higher and pH 3.5 or lower were clear at 20–80 °C, while the pH 5.0 solution was turbid. And, UCST- and LCST-type thermosensitivity was observed at 60 °C at pH 4.0 and 71 °C at pH 5.5, respectively [27]. The increased hydrophobicity of thermosensitive polymers decreased the phase transition temperature [5,6,7]. Thus, the terminal groups of PAMAM-Phe-Suc were changed from succinic acid to the more hydrophobic cyclohexyl acid. Figure 3B–F demonstrate the temperature dependencies of PAMAM-Phe-CHex bearing different Phe residues. And, the phase transition temperatures of these dendrimers at different pH values are summarized in Table 2. The PAMAM-Phe64-CHex solution at pH 7.0 was turbid at 20 °C but became clear after heating. This UCST-type thermosensitive behavior is comparable to that of PAMAM-Phe56-Suc at pH 5.5. It is because the pKa of cyclohexyl acid is higher than that of succinic acid [30]. At pH values between 6.5 and 4.5, the PAMAM-Phe64-CHex solutions were turbid, irrespective of the temperature. Before heating, the aqueous solutions of PAMAM-Phe56-Suc were clear at pH values ≤ 4.0. However, aqueous PAMAM-Phe64-CHex solutions remained turbid at pH 4.0, although the transmittance became higher. PAMAM-Phe56-Suc showed the LCST-type thermosensitive behavior, but PAMAM-Phe64-Chex did not. The hydrophobic-hydrophilic balance is important in LCST-type thermosensitive polymers [5,6,7]. PAMAM-Phe64-CHex is likely too hydrophobic to exhibit LCST-type thermosensitivity. The hydrophobicity of PAMAM-Phe-CHex increased as Phe was incorporated into the dendrimer. The UCST-type thermosensitive behavior of PAMAM-Phe46-CHex was observed at a lower phase-transition temperature at pH 7.0. The UCST of PAMAM-Phe35-CHex became lower than 20 °C at pH 7.0, and PAMAM-Phe-CHex bearing ≤ 35 Phe residues did not show the UCST under our experimental conditions. The UCST-type thermosensitive behavior of PAMAM-Phe-CHex bearing small amount of Phe was observed at lower pH, although the UCST was not in the range between 20 °C and 80 °C. When the pH of the solution was reduced to around pH 5.0, the transmittance decreased, and the UCST-type thermosensitivity diminished. However, when the pH was further decreased to 3.5, the transmittance tended to increase. Meanwhile, LCST-type thermosensitive behavior was not observed in PAMAM-Phe-CHex bearing ≥35 Phe residues. LCST-type thermosensitive behavior was observed in PAMAM-Phe-CHex bearing ≤27 Phe residues, but the phase transition became broad. Overall, controlling the phase transition temperature of the dendrimer proved difficult for carboxylate-terminal Phe-modified dendrimers.

**Figure 4:**
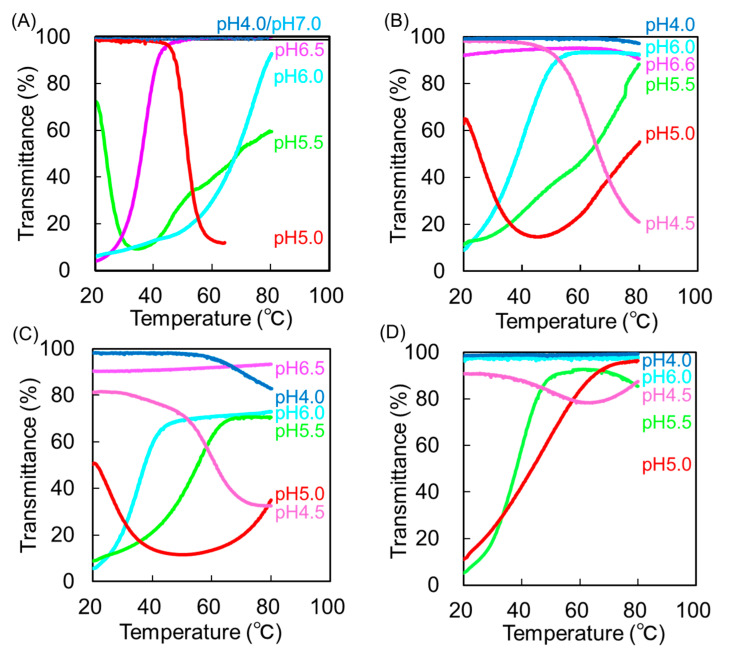
Temperature-dependent transmittance curves of aqueous solutions containing PAMAM-Phe-SO_3_Na with different Phe/PAMAM ratios at different pH values (1 mg/mL). (**A**) PAMAM-Phe57-SO_3_Na64, (**B**) PAMAM-Phe37-SO_3_Na68, (**C**) PAMAM-Phe27-SO_3_Na61, and (**D**) PAMAM-Phe16-SO_3_Na62.

Next, we examined the temperature-dependent transmittance of PAMAM-Phe-SO_3_Na with Phe/PAMAM ratios at varying pH values (Figure 4). The PAMAM-Phe57-SO_3_Na64 solution was clear at pH 7.0 and 4.0, irrespective of the temperature. UCST- and LCST-type thermosensitive behaviors were observed at 36 °C at pH 6.5 and 50 °C at pH 5.0, respectively. Both UCST- and LCST-type phase transitions at 24 °C and 68 °C were observed at pH 5.5 [27]. As the bound number of Phe decreased, the UCST and the solution became lower and clearer, respectively. As the bound number of Phe decreased, the LCST was observed at lower pH. pH-Switchable UCST- and LCST-type thermosensitive behaviors were observed in these dendrimer solutions, except for PAMAM-Phe16-SO_3_Na62. The phase transition temperatures of these dendrimers at different pH values are summarized in Table 3. PAMAM-Phe-SO_3_Na, with fewer Phe residues, demonstrated a lower UCST at pH 5.5 and pH 6 but also a lower LCST at pH 5. The sensitivity at body temperature could be changed from pH 6.5 to 5.5 by varying the Phe/PAMAM ratio. Thus, the Phe/PAMAM ratio facilitated the control over the sensitivity of PAMAM-Phe-SO_3_Na, which is useful for biomedical applications.

## 4. Discussion

We previously developed unique pH-switchable dendrimers (PAMAM-Phe-Suc and PAMAM-Phe-SO_3_Na) with LCST- and UCST-type thermosensitive behaviors [27]. Some LCST-type thermosensitive polymers, such as PNIPAM, are known to adopt UCST-type behavior upon changing the solvent [9,10,11]. The pH-switchable thermosensitive behavior of our dendrimers is unique. In this study, we attempted to regulate the stimuli-responsive properties of anionic terminal Phe-modified dendrimers by varying the structure of the terminal group and the Phe/PAMAM ratio. We first investigated the phase transitions of various carboxylate-terminal Phe-modified dendrimers at different pH values. Since PAMAM-Phe-Suc exhibits the LCST-type phase transition at 71 °C, dendrimers with increased hydrophobicity were designed and synthesized to decrease the LCST. However, the thermosensitive properties of the dendrimers diminished upon conversion of the dendrimer termini from succinic acid to cyclohexyl acid, negating its previous LCST-type thermosensitive behavior. This could be due to the extremely increased hydrophobicity of cyclohexyl acid. Then, dendrimers with fewer Phe residues were synthesized to decrease the hydrophobicity. The UCST of dendrimers with fewer Phe residues became lower at pH 7.0, but the UCST-type phase transition of PAMAM-Phe-CHex with 35 or less Phe residues was not observed in the range from 20 °C to 80 °C. Although LCST-like thermosensitivity was observed for PAMAM-Phe-CHex with 27 or fewer Phe residues, there was no obvious phase transition. As a result, adjusting the phase transition temperature, by altering the dendrimer structure of carboxylate-terminal Phe-modified dendrimers, was difficult.

We then attempted to control the phase transition temperature of the sulfonate-terminal Phe-modified dendrimers (PAMAM-Phe-SO_3_Na). PAMAM-Phe-SO_3_Na with different Phe/PAMAM ratios were synthesized, and its phase transition behaviors were analyzed. Lower LCST and UCST were observed for PAMAM-Phe-SO_3_Na with fewer Phe residues at the same pH. Thus, changing the dendrimer structure could regulate the phase transition of sulfonate-terminal Phe-modified dendrimers. A sharper thermosensitivity was observed for sulfonate-terminal Phe-modified dendrimers than those for carboxylate-terminal Phe-modified dendrimers. This may be due to the zwitterionic structure at every terminal with a stable anion derived from the sulfonate. The phase transition temperature was also affected by the pH. Higher LCST and UCST were observed at lower pH for the same PAMAM-Phe-SO_3_Na dendrimer. Thus, PAMAM-Phe-SO_3_Na dendrimers can be applicable as smart materials.

**Figure 5:**
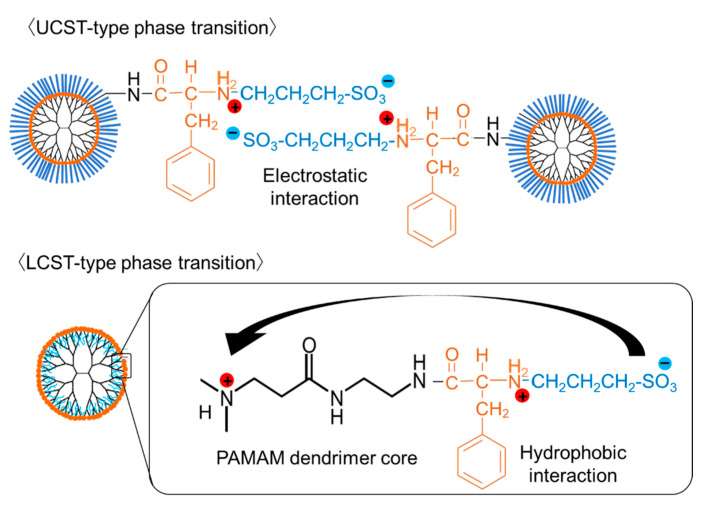
Possible phase transition mechanisms of PAMAM-Phe-SO_3_Na.

Our previous paper indicated that coacervated droplets were observed when the transmittance of the solutions containing PAMAM-Phe-Suc and PAMAM-Phe-SO_3_Na became low [27]. Dendrimers synthesized in this study were probably coacervated when the transmittance was low. The phase transition mechanism of this kind of dendrimer is discussed. The ionic effects in the phase transition of anionic terminal fully Phe-modified dendrimers were previously investigated [27]. The dendrimer solutions, which showed the UCST-and LCST-type thermosensitivity, became clear and turbid by adding NaCl, indicating that both LCST- and UCST-type thermosensitive behaviors disappeared in the presence of NaCl. In the presence of salt, the intermolecular electrostatic interactions between zwitterionic groups are suppressed [5], and hydrophobic interaction becomes strong [31]. These facts suggest that electrostatic interaction and hydrophobic interactions play important roles to the UCST- and LCST-type thermosensitive behaviors of these dendrimers, respectively [27], as shown in Figure 5. Our previous paper also indicated that the ζ-potential of these dendrimers was changed from negative to positive as the pH decreased [27]. The negative surfaces increased the dendrimer solubility. When the ζ-potential became neutral, the zwitterionic dendrimers were associated each other via the electrostatic interaction, which induced the UCST-type phase transition (Figure 5). When the ζ-potential became positive, the anionic termini of the dendrimers were not located at the surface. It is possible that the anionic termini were interacted with tertiary amines of the PAMAM dendrimer core. Consequently, Phe residues were located at the dendrimer surface, which possibly induced the LCST-type phase transition based on the hydrophobic interaction (Figure 5). We synthesized sulfonate-terminal Phe-modified dendrigraft-polylysine without any tertiary amines and examined the temperature-dependent transmittance at different pHs. Figure S23 shows a UCST-type thermosensitive behavior at pH 7, but not any LCST-type thermosensitive behaviors. This suggests that the core PAMAM dendrimer is important for pH-switchable LCST/UCST-type thermosensitive behaviors. In this study, the influence of the hydrogen bond on the phase transition behaviors was also investigated. The temperature-dependent transmittance of these dendrimers in heavy water was measured. Interestingly, carboxylate-terminal PAMAM-Phe-Suc showed higher LCST and lower UCST in heavy water, but sulfonate-terminal PAMAM-Phe-SO_3_Na showed lower LCST and higher UCST (Figure S24). This suggests that the effect of hydrogen bonding in PAMAM-Phe-SO_3_Na is different from that in PAMAM-Phe-Suc. Besides this, we examined the effect of the dendrimer concentration to the phase transition behaviors. Figure S25 shows that the UCST and the LCST of these dendrimers were almost unchanged by the concentration under our conditions. However, the initial solubility of PAMAM-Phe-CHex was greatly changed by lowering the dendrimer concentration. The detailed phase transition mechanisms remain to be investigated.

In conclusion, we attempted to regulate the stimuli-sensitive behaviors of pH-switchable LCST/UCST-type thermosensitive dendrimers. This was done by varying the terminal group and the Phe/PAMAM ratio in carboxylate- and sulfonate-terminal dendrimers. Although varying the terminal group and the Phe/PAMAM ratio for carboxylate-terminal Phe-modified dendrimers did not show any clear phase transition, the phase transition temperature of PAMAM-Phe-SO_3_Na increased when the Phe/PAMAM ratio decreased. The ability to regulate the sensitivity of polymers to various stimuli will facilitate their utilization in many applications.

## Figures and Tables

**Table 1:** List of synthesized dendrimers in this study.

Dendrimer ^1^	Phe		Suc/CHex/SO_3_Na	Thermo- Sensitivity
	In Feed	Bound	Bound	
PAMAM-Phe-Suc ^2^	96	56	57	LCST/UCST
PAMAM-Phe64-CHex	96	64	62	UCST
PAMAM-Phe46-CHex	62	46	~64	UCST
PAMAM-Phe35-CHex	46	35	~64	UCST
PAMAM-Phe27-CHex	38	27	62	LCST/UCST
PAMAM-Phe16-CHex	27	16	~64	LCST/UCST
PAMAM-Phe57-SO3Na64 ^2^	96	57	64	LCST/UCST
PAMAM-Phe37-SO3Na68	49	37	68	LCST/UCST
PAMAM-Phe27-SO3Na61	38	27	61	LCST/UCST
PAMAM-Phe16-SO3Na62	27	16	62	UCST

^1^ The number of terminal groups in the dendrimer is 64. ^2^ Referred to our previous reports [27,28].

**Table 2:** pH-switchable thermosensitive behavior of PAMAM-Phe-CHex at different pH values ^1^.

Dendrimer	Temperature (°C)			
	pH 7	pH 5	pH 4.5	pH 4
PAMAM-Phe64-CHex	60 (U)	Turbid	Turbid	Slightly
				turbid
PAMAM-Phe46-CHex	46 (U)	>80 (U)	Turbid	Slightly
				turbid
PAMAM-Phe35-CHex	<20 (U)	>80 (U)	Turbid	Slightly
				turbid
PAMAM-Phe27-CHex	Clear	Turbid	32 (L)	Clear
PAMAM-Phe16-CHex	Clear	>80 (U)	68 (L)	Clear

^1^ Phase transition temperatures (UCST and LCST) were estimated as the temperatures with 50% transmittance, which are shown as (U) and (L), respectively.

**Table 3:** pH-switchable thermosensitive behavior of PAMAM-Phe-SO_3_Na at different pH values ^1^.

Dendrimer	Temperature (°C)				
	pH 6.5	pH 6	pH 5.5	pH 5	pH 4
PAMAM-Phe57-SO_3_Na64	36 (U)	68 (U)	24 (L)/	50 (L)	Clear
			68 (U)		
PAMAM-Phe37-SO_3_Na68	Clear	38 (U)	62 (U)	25 (L)/	Clear
				76 (U)	
PAMAM-Phe27-SO_3_Na61	Clear	37 (U)	55 (U)	21 (L)/	Clear
				>80 (U)	
PAMAM-Phe16-SO_3_Na62	Clear	Clear	38 (U)	44 (U)	Clear

^1^ Phase transition temperatures (UCST and LCST) were estimated as the temperatures with 50% transmittance, which are shown as (U) and (L), respectively.

## Data Availability

The data supporting the findings of this study are available from the corresponding author upon reasonable request.

## Supplementary Materials

The following supporting information can be downloaded at https://www.mdpi.com/article/10.3390/polym14122426/s1, Figure S1: The dendrimer structure for the peak assignment. Figures S2–S21: ^1^H NMR spectra of the dendrimers synthesized in this study. Figure S22: FT-IR spectra of the dendrimers synthesized in this study. Figures S23–S25. Temperature-dependent transmittance curves of dendrigraft polylysine in different pHs, PAMAM-Phe-Suc and PAMAM-Phe-SO_3_Na in H_2_O and D_2_O buffers, and PAMAM-Phe-CHex and PAMAM-Phe-SO_3_Na at different dendrimer concentrations.
