# Supplementary material for: Control of Stimuli Sensitivity in pH-Switchable LCST/UCST-Type Thermosensitive Dendrimers by Changing the Dendrimer Structure
Source: Polymers (Basel). 2022 Jun 15;14(12):2426. doi: 10.3390/polym14122426 (PMC9227611; doi:10.3390/polym14122426)
Supplement: Supplementary file 1 [file polymers-14-02426-s001.zip › polymers-1727518-supplementary.pdf]

Supplementary Information

Control of Stimuli Sensitivity in pH-Switchable LCST/UCST-type Thermosensitive Dendrimers

Chie Kojima[1,]* , Yunshen Fu[1], and Mamiko Tamaki[1]

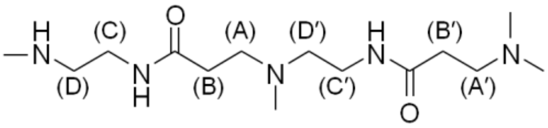

Figure S1. Partial dendrimer structure for assignment of the following ^{1}H NMR spectra.

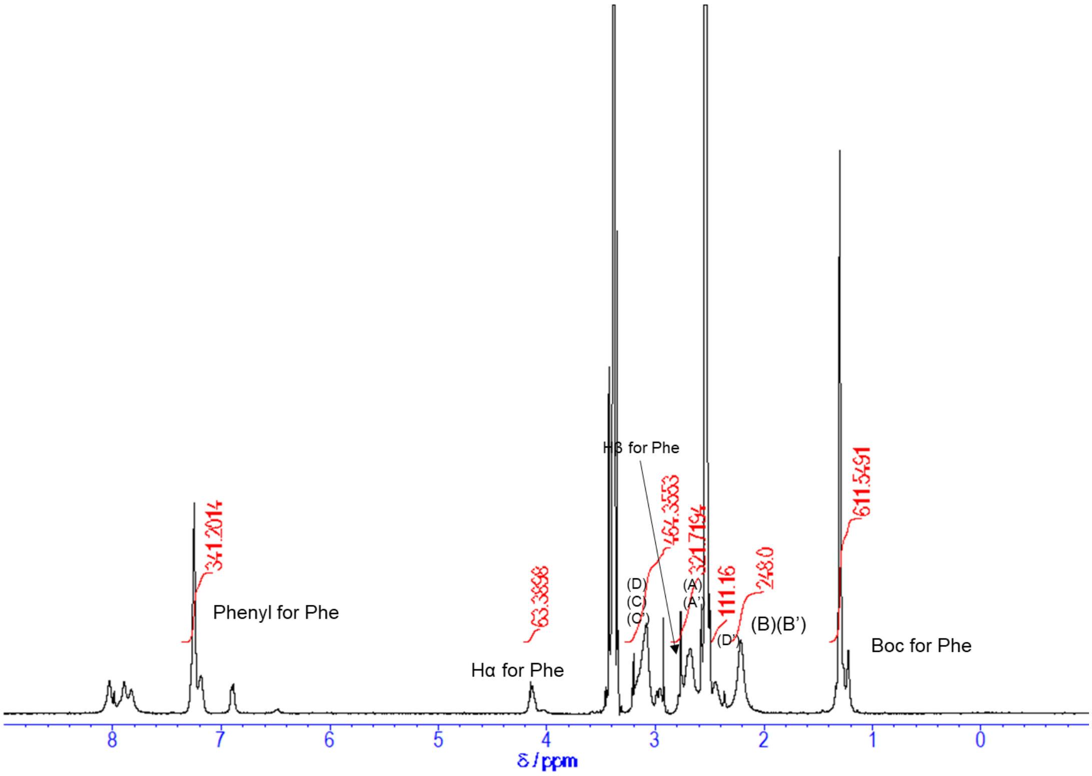

Figure S2. ^{1}H NMR spectrum of PAMAM-(Boc-Phe)64 in DMSO-d_6.

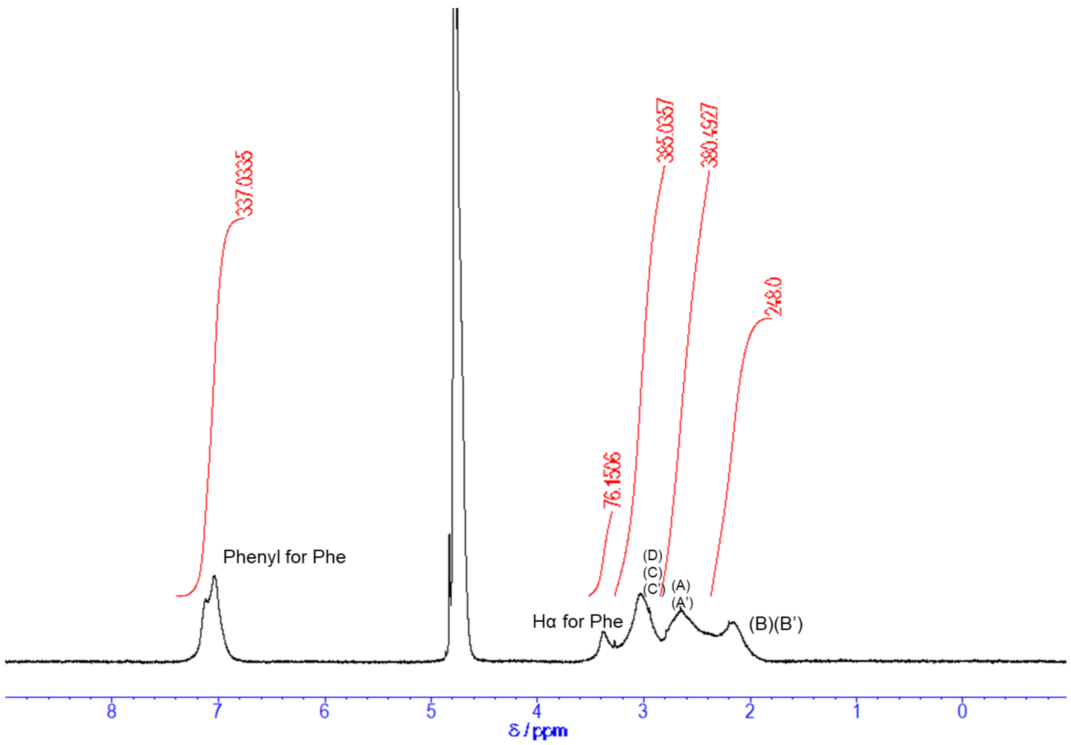

Figure S3. ^{1}H NMR spectrum of PAMAM-Phe64 in D_2O.

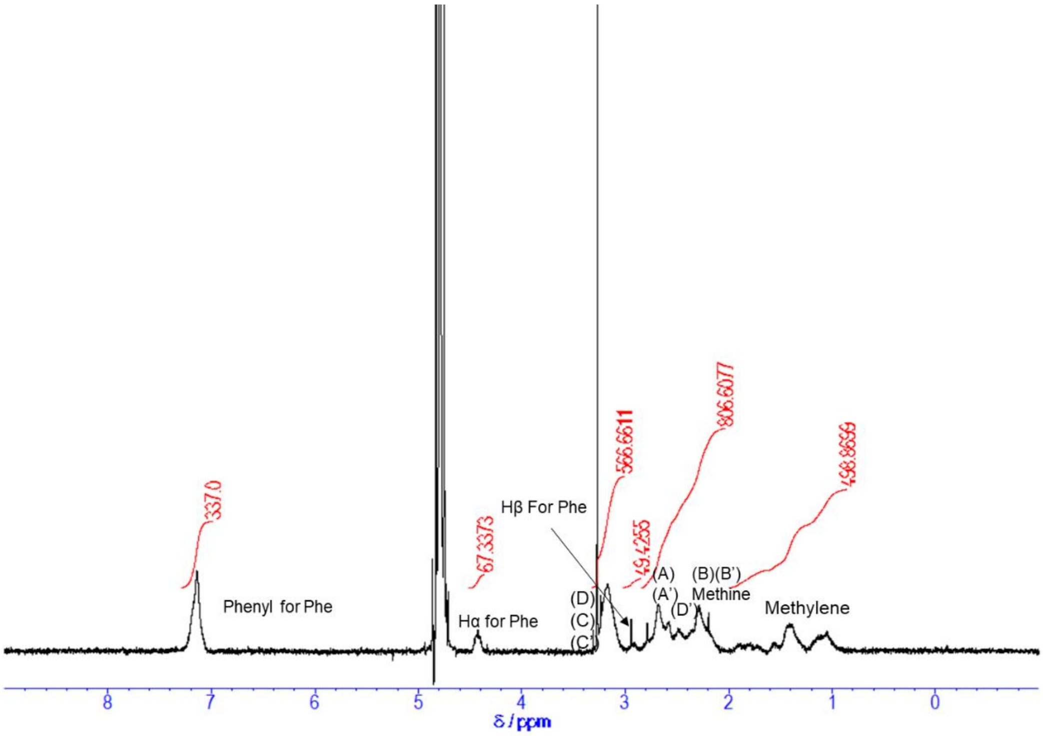

Figure S4. ^{1}H NMR spectrum of PAMAM-Phe64-CHex in D_2O containing NaOD.

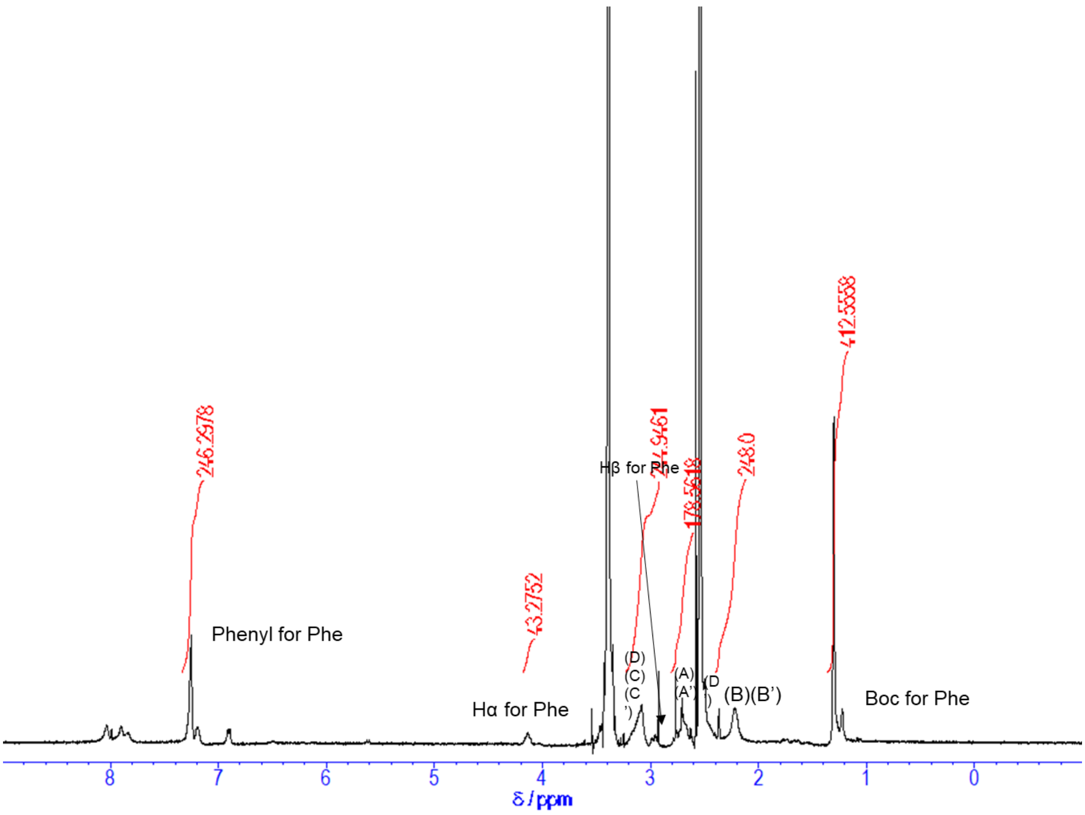

Figure S5. ^{1}H NMR spectrum of PAMAM-(Boc-Phe)46 in DMSO-d_6.

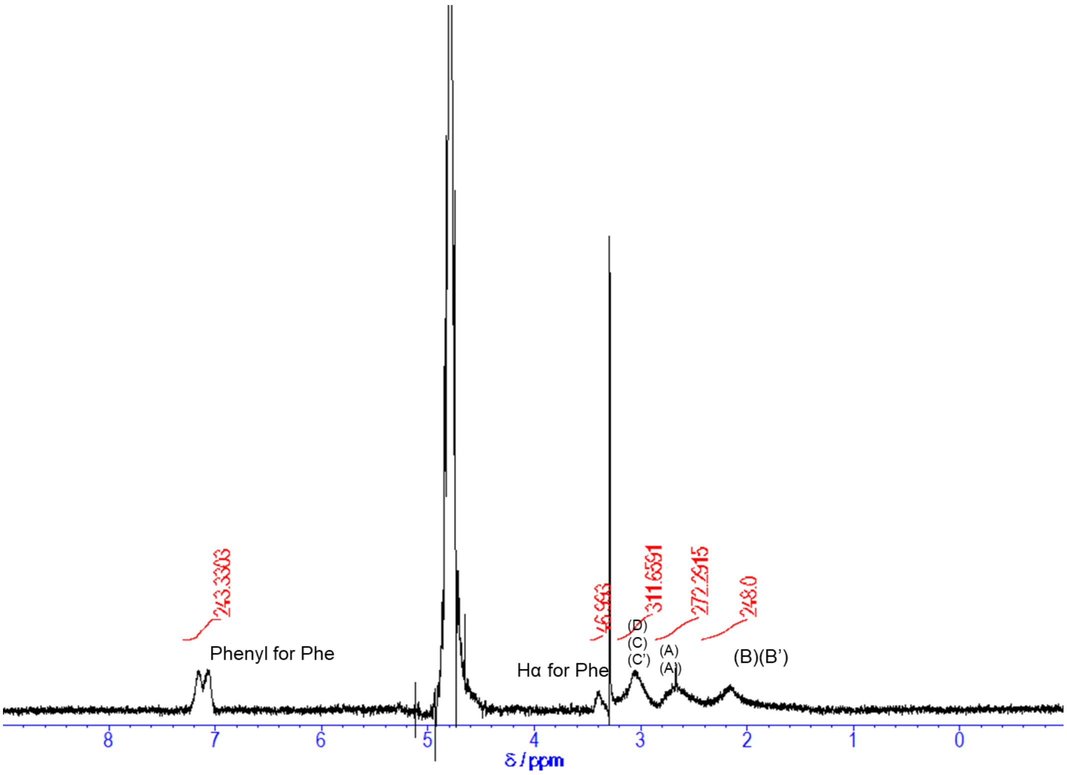

Figure S6. ^{1}H NMR spectrum of PAMAM-Phe46 in D_2O.

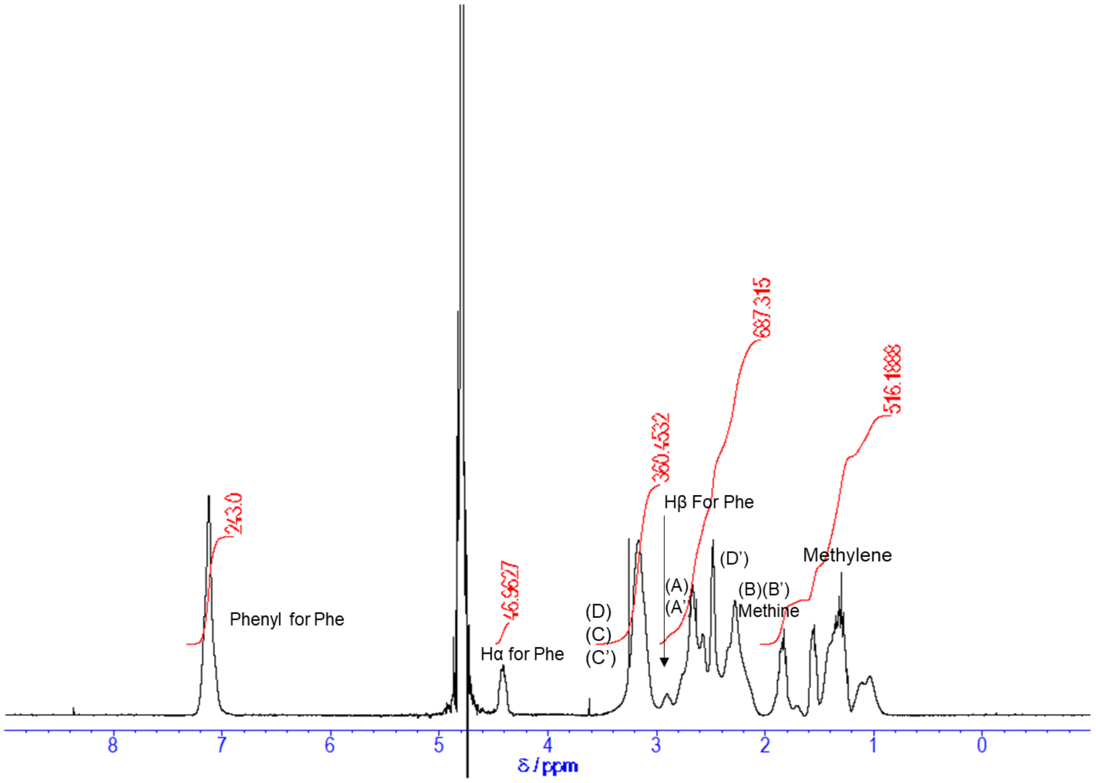

Figure S7. ^{1}H NMR spectrum of PAMAM-Phe46-CHex in D_2O containing NaOD.

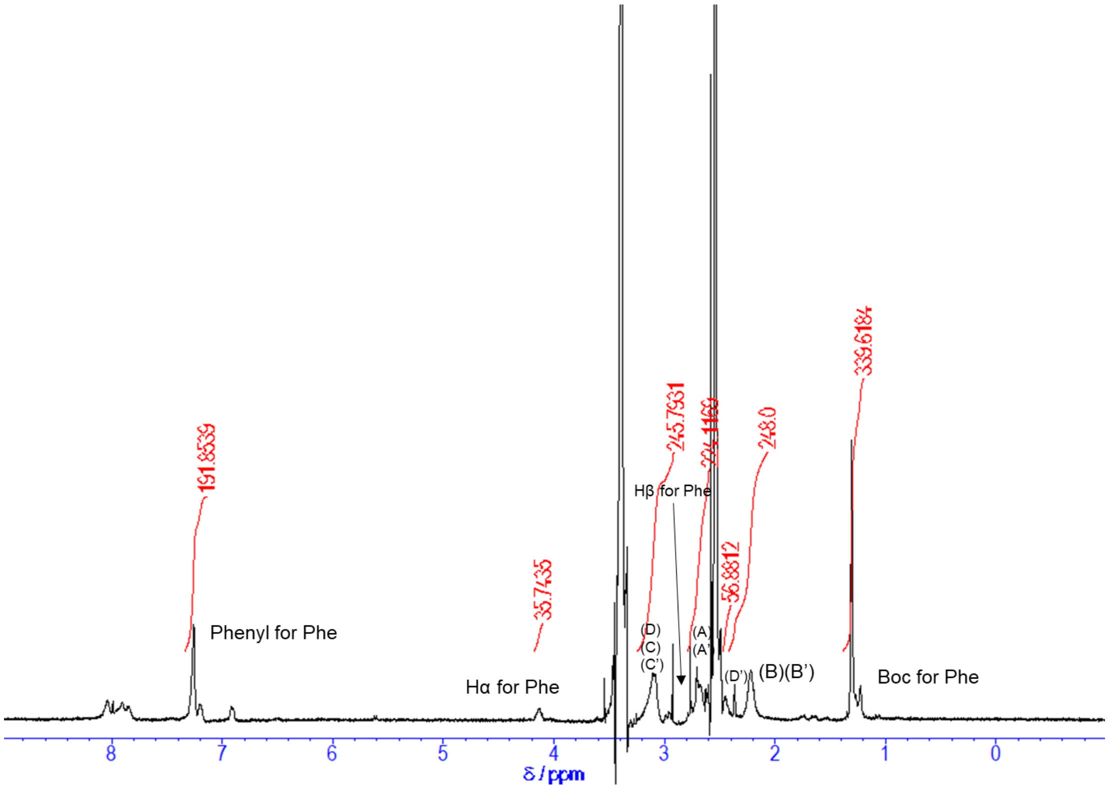

Figure S8. ^{1}H NMR spectrum of PAMAM-(Boc-Phe)35 in DMSO-d_6.

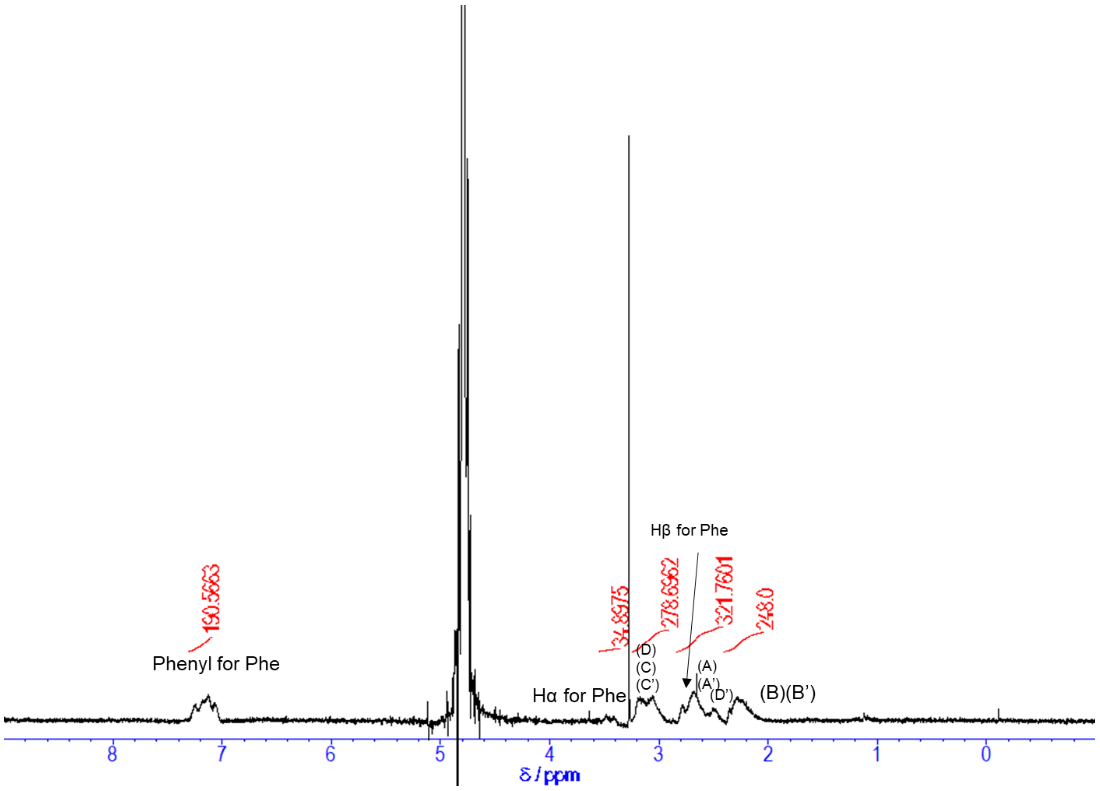

Figure S9. ^{1}H NMR spectrum of PAMAM-Phe35 in D_2O.

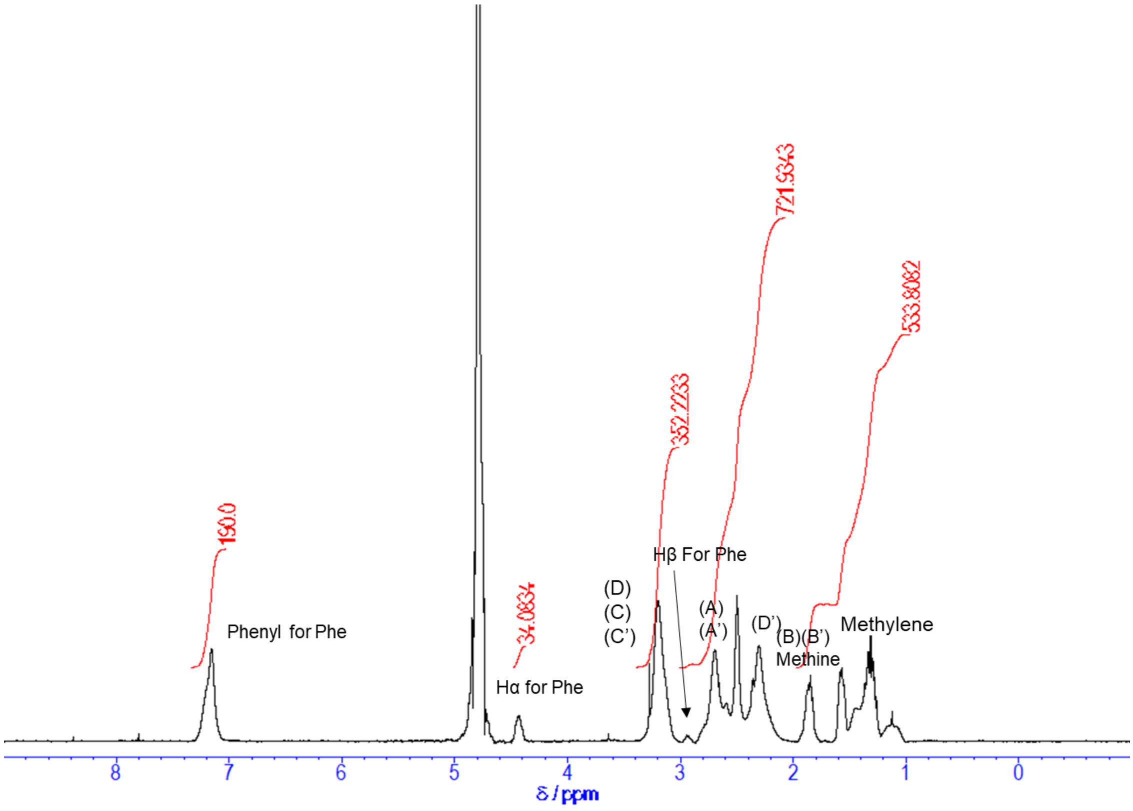

Figure S10. ^{1}H NMR spectrum of PAMAM-Phe35-CHex in D_2O containing NaOD.

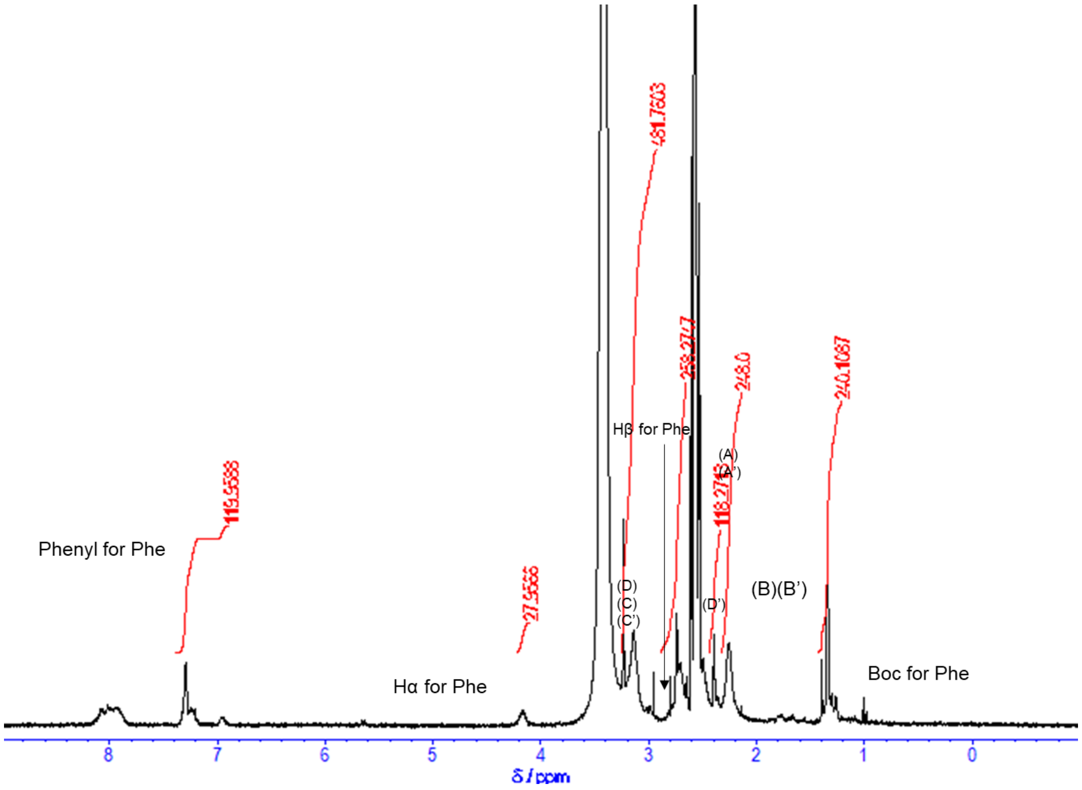

Figure S11. ^{1}H NMR spectrum of PAMAM-(Boc-Phe)27 in DMSO-d_6.

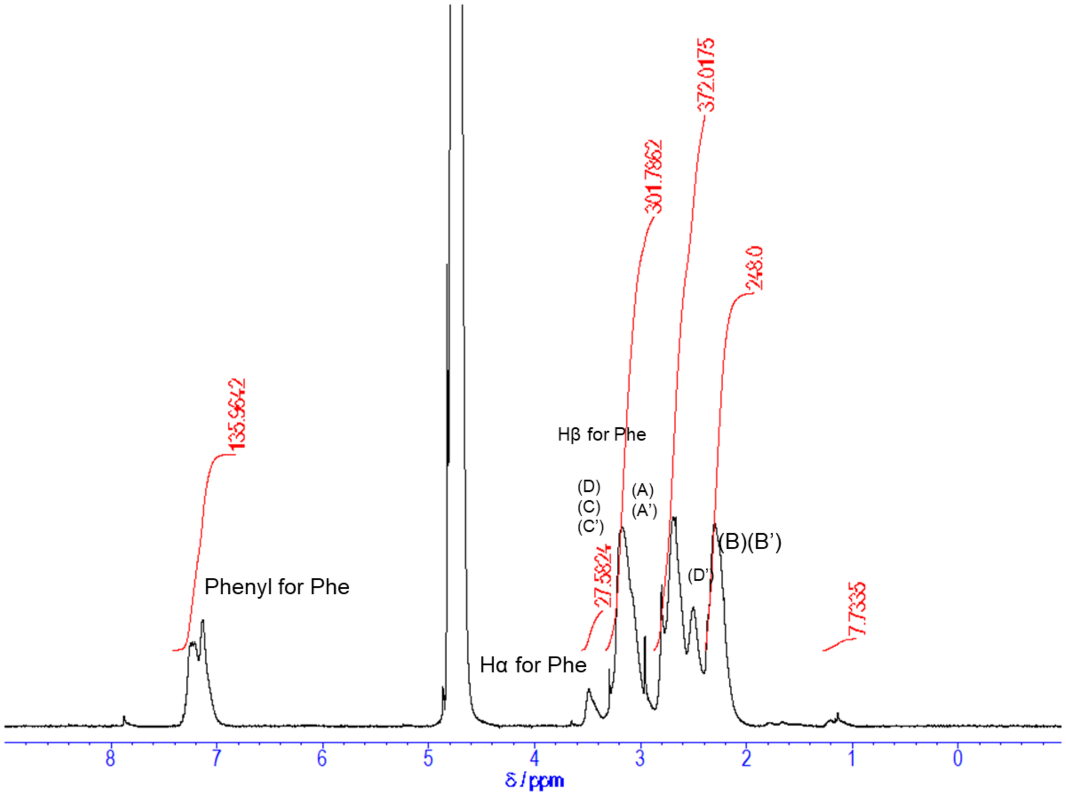

Figure S12. ^{1}H NMR spectrum of PAMAM-Phe27 in D_2O.

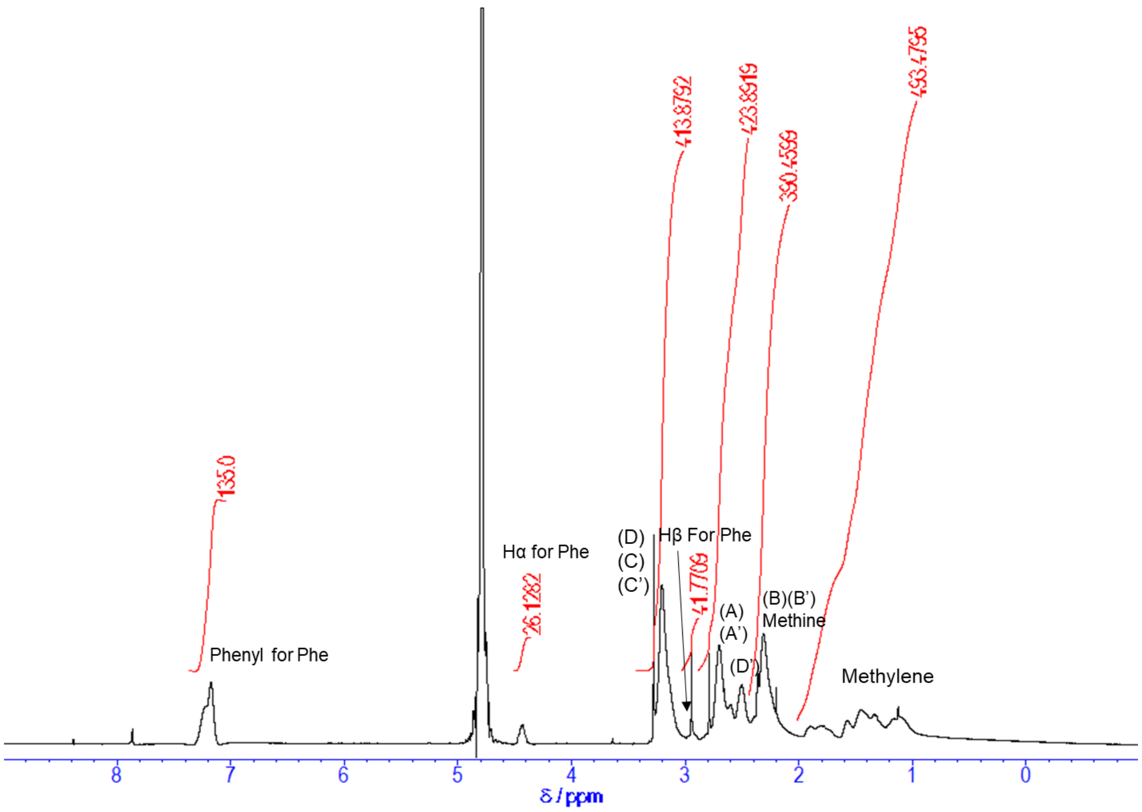

Figure S13. ^{1}H NMR spectrum of PAMAM-Phe27-CHex in D_2O containing NaOD.

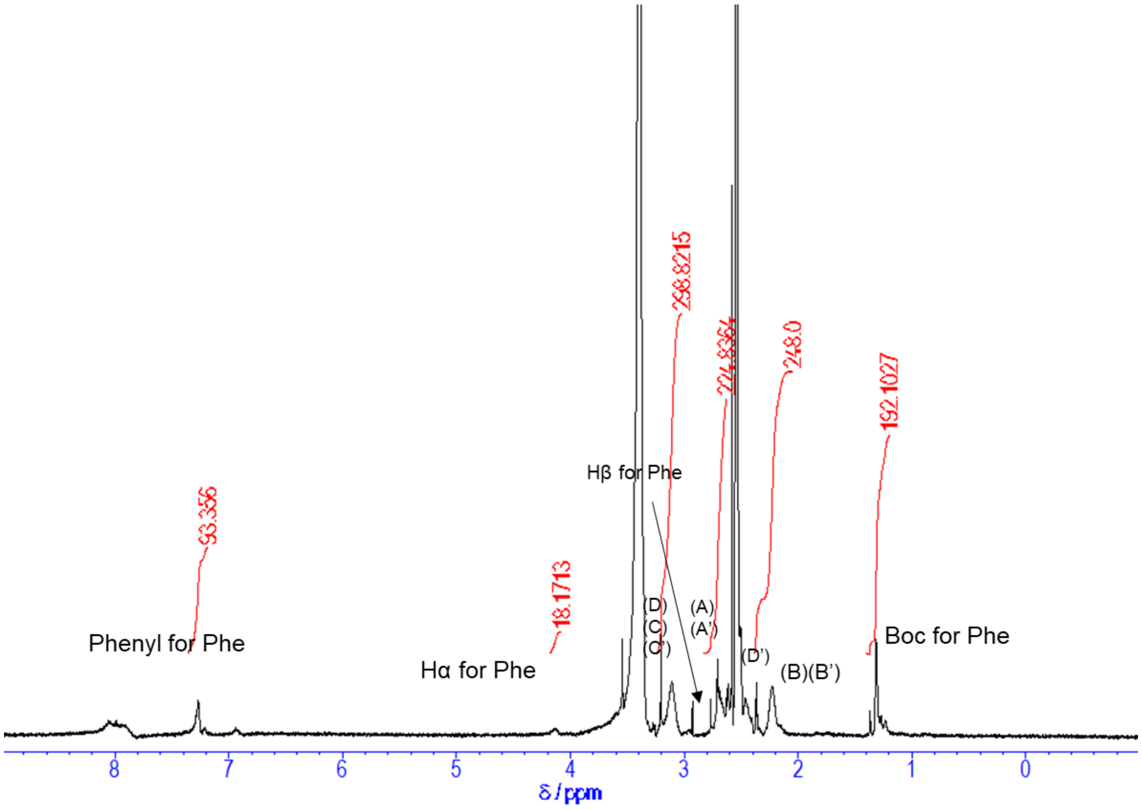

Figure S14. ^{1}H NMR spectrum of PAMAM-(Boc-Phe)16 in DMSO-d_6.

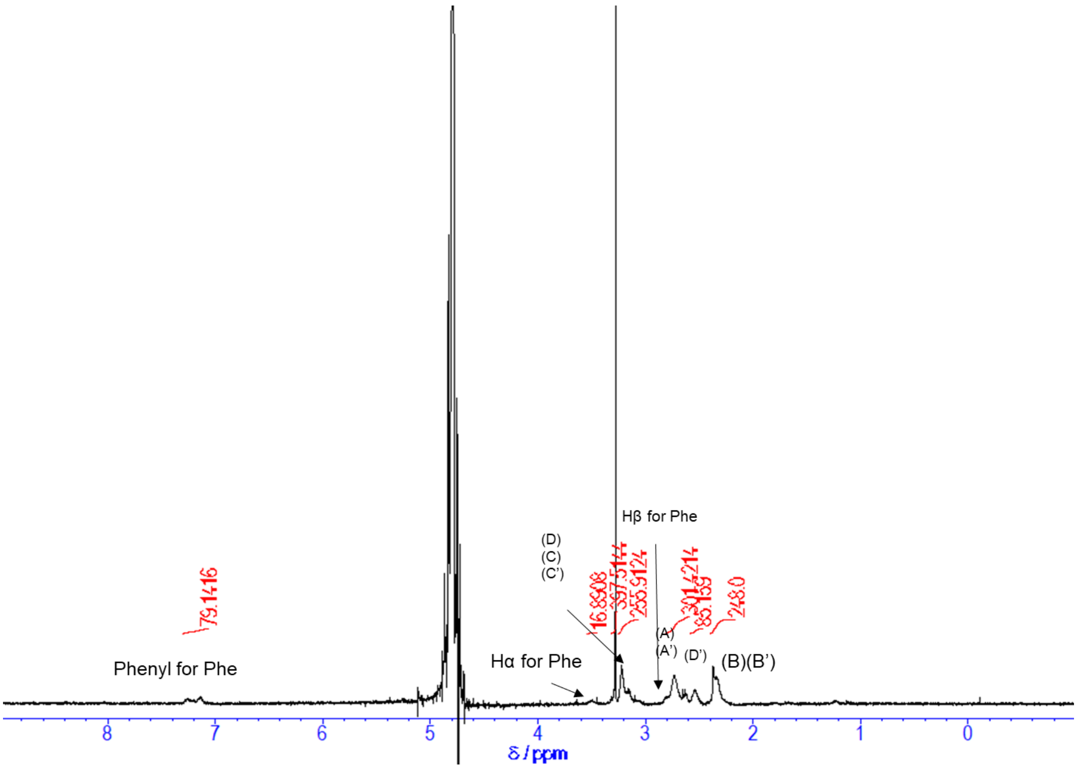

Figure S15. ^{1}H NMR spectrum of PAMAM-Phe16 in D_2O.

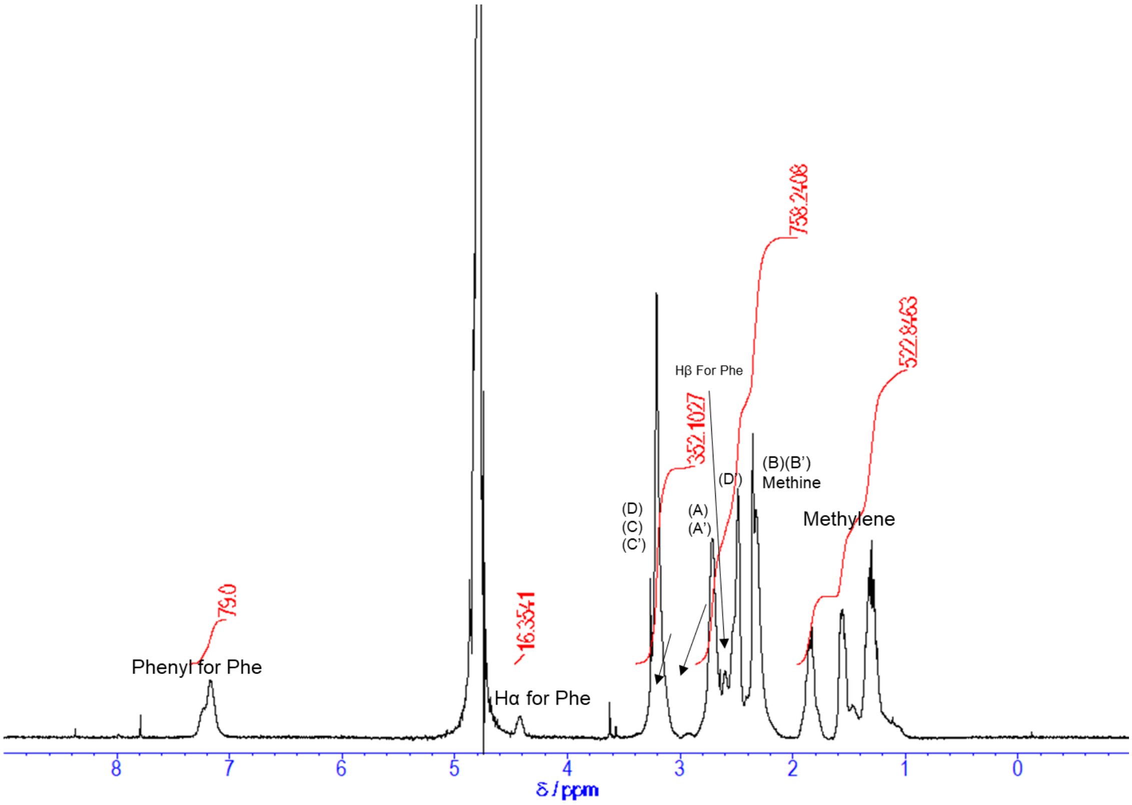

Figure S16. ^{1}H NMR spectrum of PAMAM-Phe16-CHex in D_2O containing NaOD.

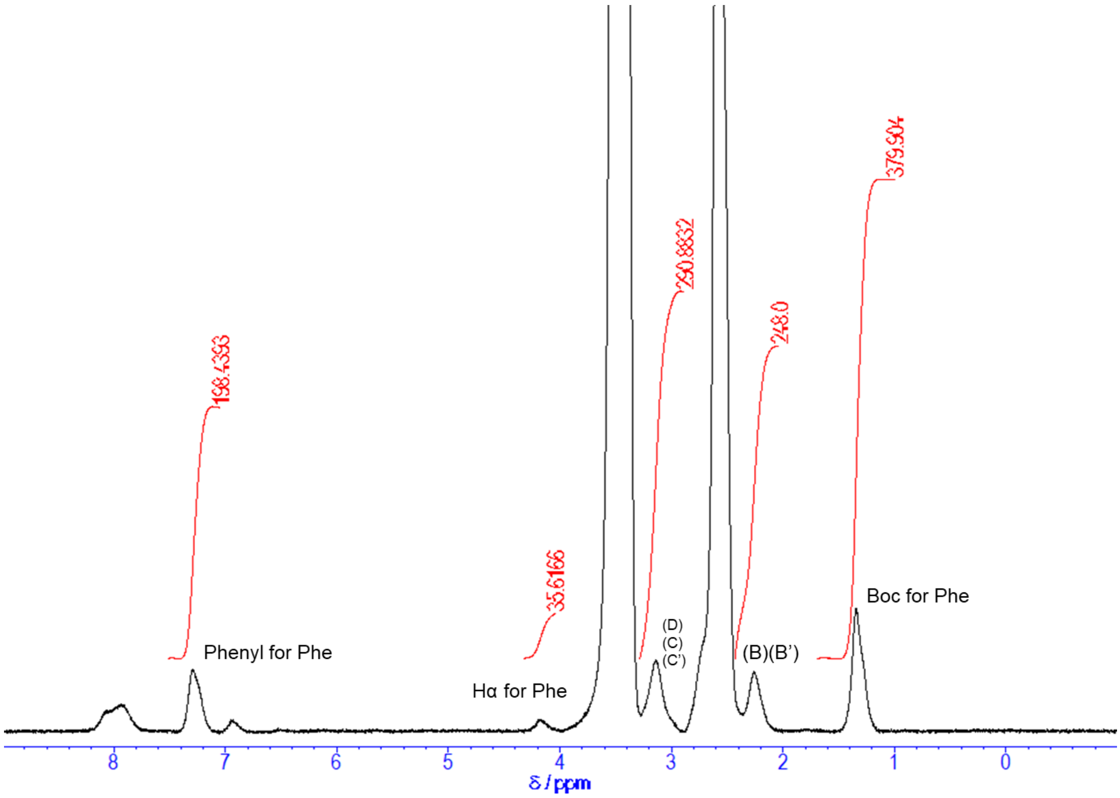

Figure S17. ^{1}H NMR spectrum of PAMAM-(Boc-Phe)37 in DMSO-d_6.

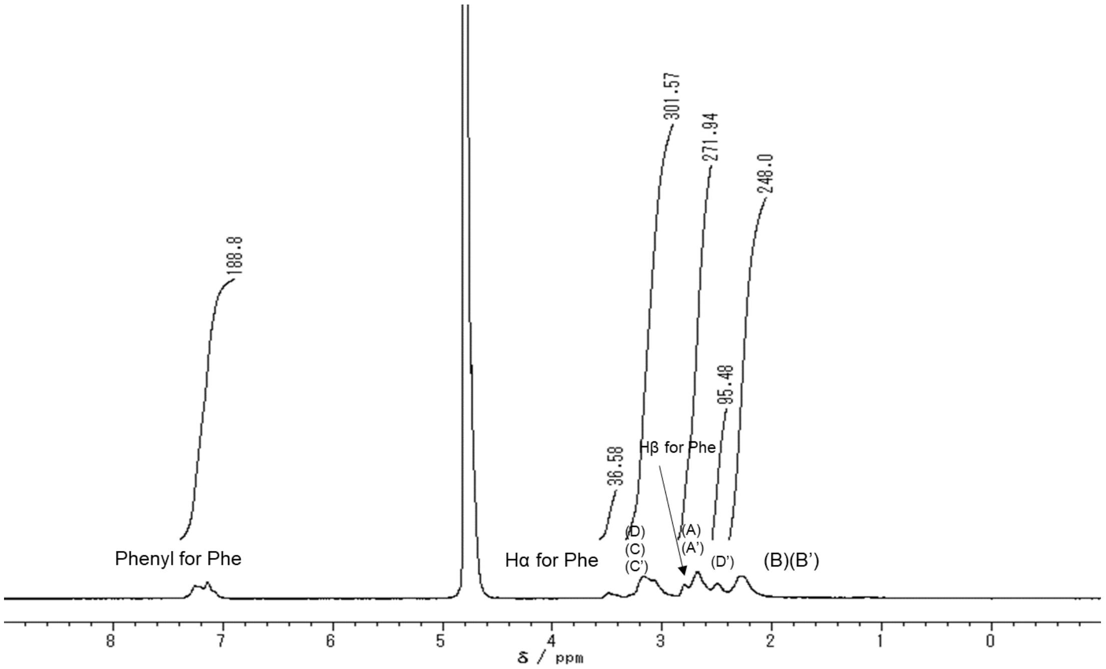

Figure S18. ^{1}H NMR spectrum of PAMAM-Phe37 in D_2O.

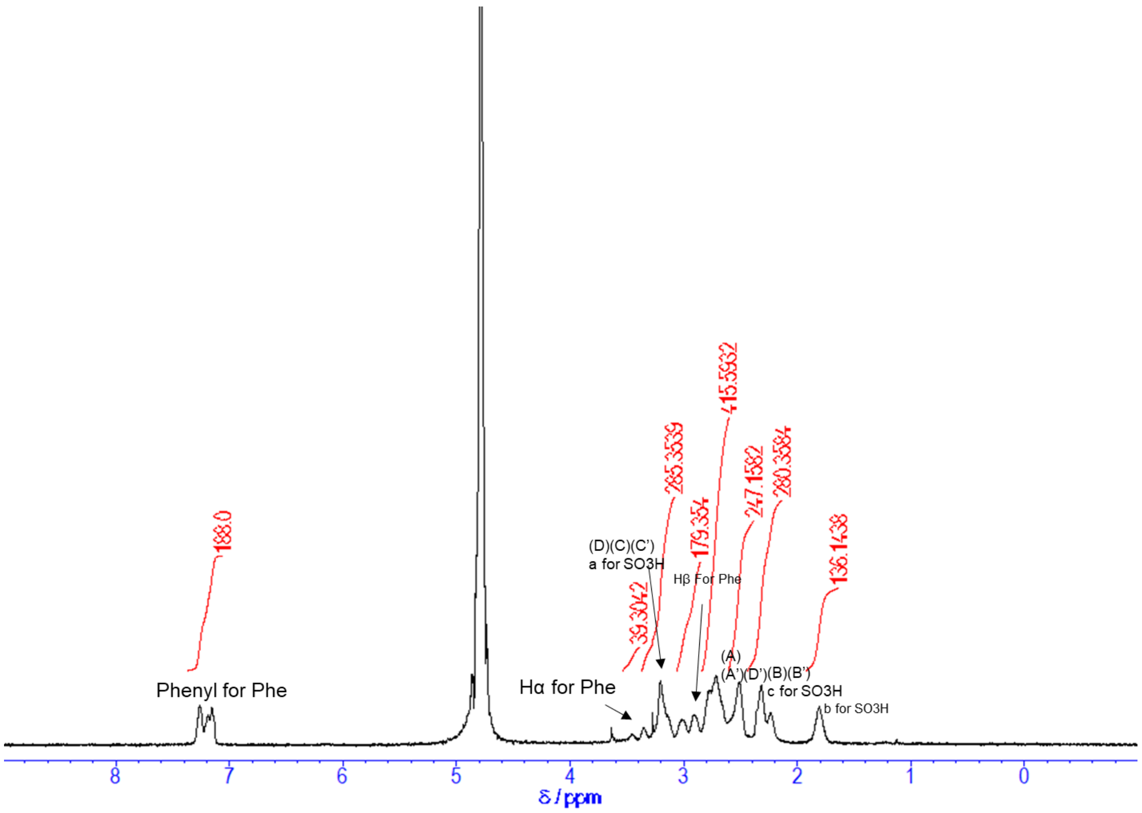

Figure S19. ^{1}H NMR spectrum of PAMAM-Phe37-SO_3Na68 in D_2O containing NaOD.

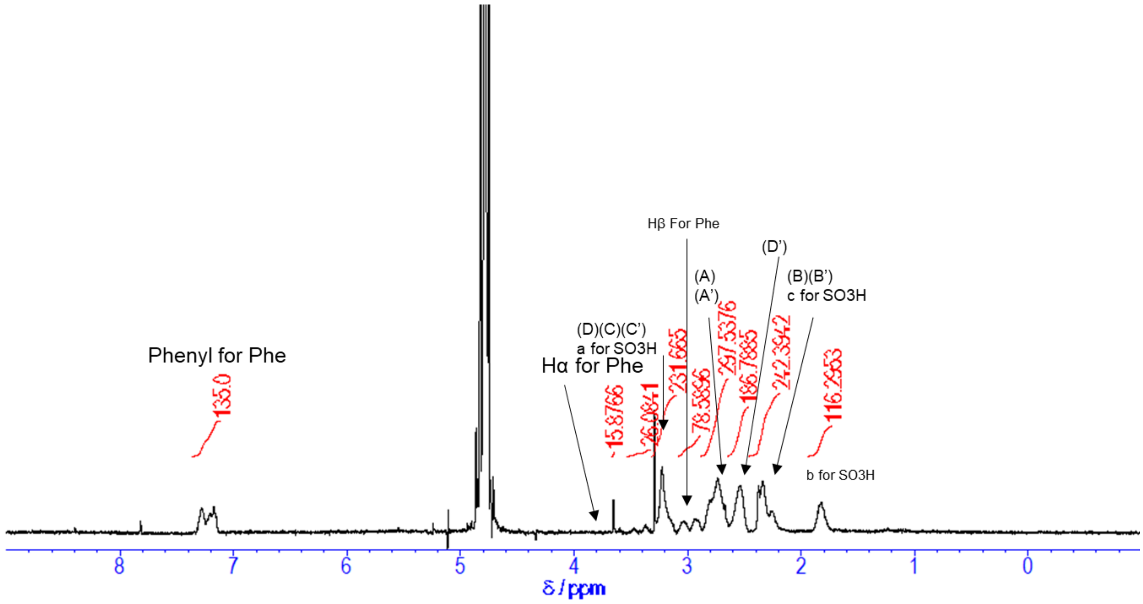

Figure S20. ^{1}H NMR spectrum of PAMAM-Phe27-SO_3Na61 in D_2O containing NaOD.

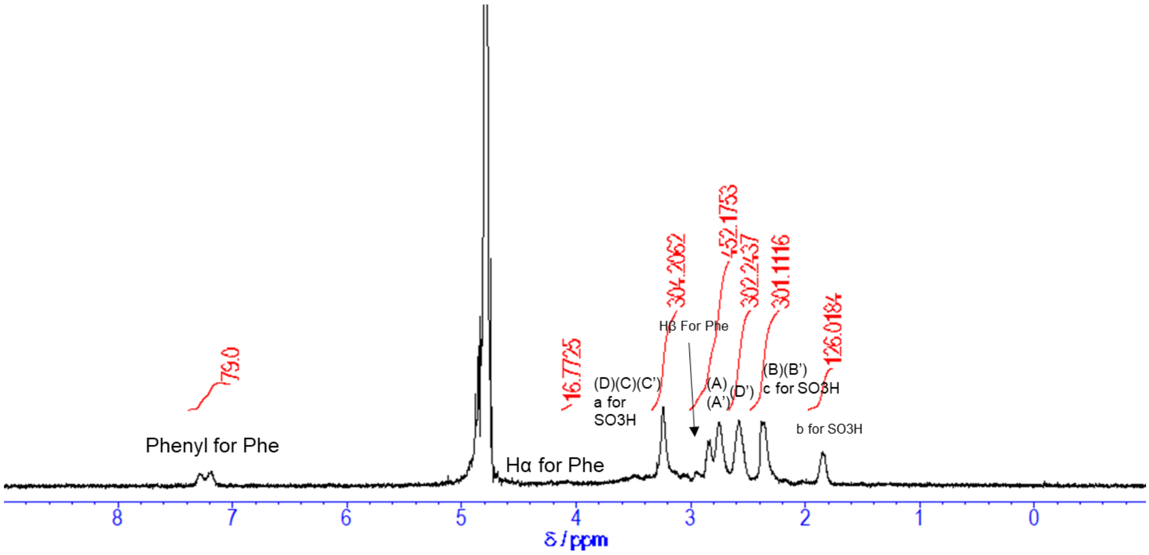

Figure S21. ^{1}H NMR spectrum of PAMAM-Phe16-SO_3Na62 in D_2O containing NaOD.

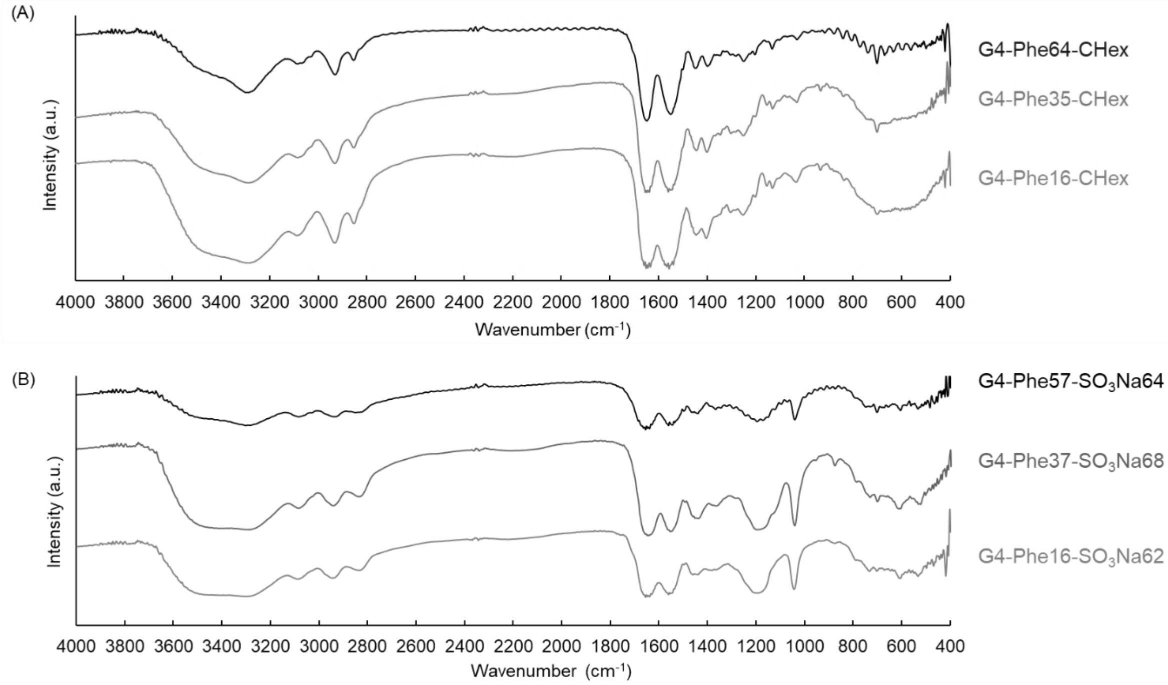

Figure S22. FT-IR spectra of PAMAM-Phe-CHex (A) and PAMAM-Phe-SO_3Na (B).

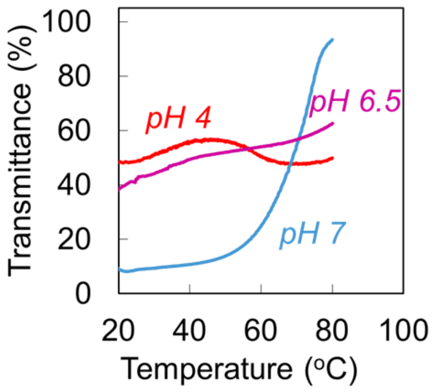

Figure S23. Temperature-dependent transmittance curves of dendrigraft polylysine (G3, 123 termini)-Phe123-SO_3Na85 in different pHs (1 mg/mL).

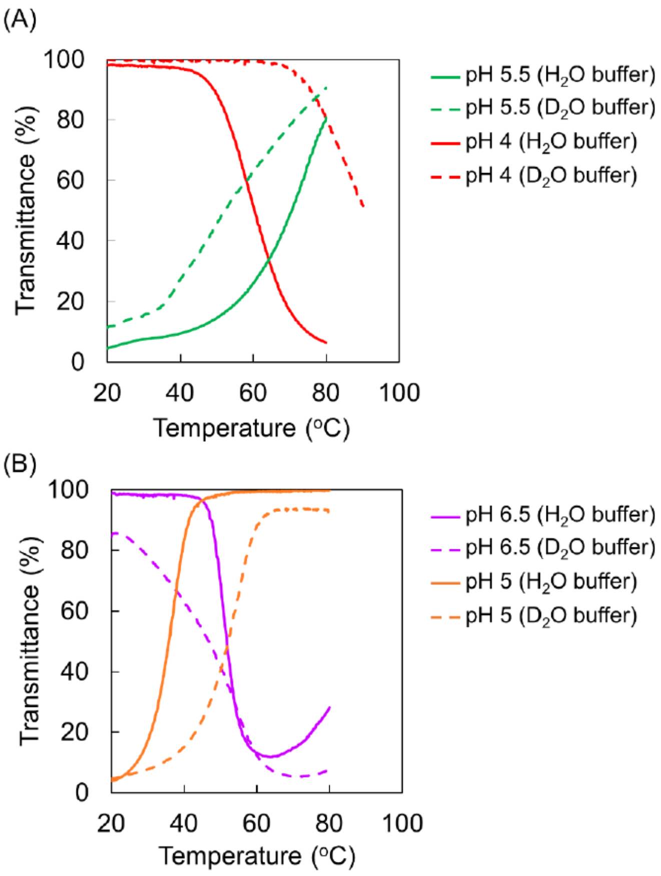

Figure S24. Temperature-dependent transmittance curves of PAMAM-Phe-Suc (A) and PAMAM-Phe-SO_3Na (B) in H_2O and D_2O buffers (1 mg/mL).

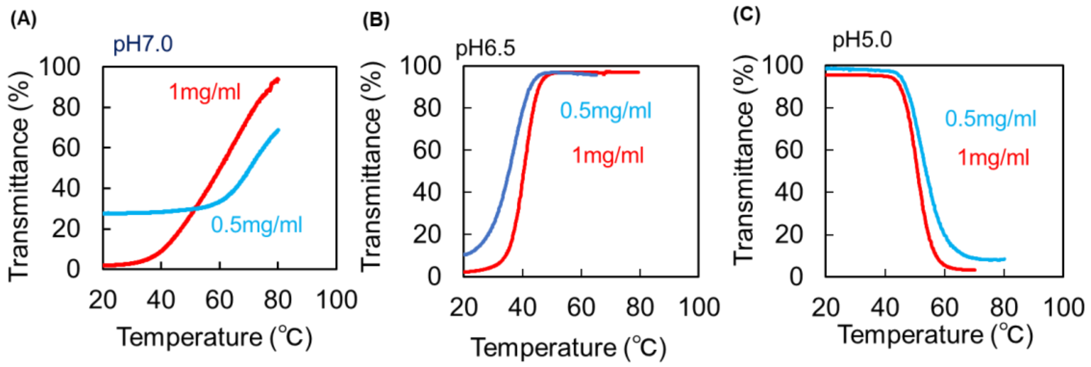

Figure S25. Temperature-dependent transmittance curves of PAMAM-Phe-CHex (A) and PAMAM-Phe-SO_3Na (B,C) at different dendrimer concentrations in different pHs.
